# Loss of Hippocampal Neurons after Kainate Treatment Correlates with Behavioral Deficits

**DOI:** 10.1371/journal.pone.0084722

**Published:** 2014-01-07

**Authors:** Gisela H. Maia, José L. Quesado, Joana I. Soares, Joana M. do Carmo, Pedro A. Andrade, José P. Andrade, Nikolai V. Lukoyanov

**Affiliations:** 1 Departamento de Anatomia, Faculdade de Medicina da Universidade do Porto, Porto, Portugal; 2 Neural Networks Group, Instituto de Biologia Molecular e Celular da Universidade do Porto, Porto, Portugal; 3 Programa Doutoral em Neurociências, Universidade do Porto, Porto, Portugal; Consejo Superior de Investigaciones Cientificas - Instituto Cajal, Spain

## Abstract

Treating rats with kainic acid induces status epilepticus (SE) and leads to the development of behavioral deficits and spontaneous recurrent seizures later in life. However, in a subset of rats, kainic acid treatment does not induce overt behaviorally obvious acute SE. The goal of this study was to compare the neuroanatomical and behavioral changes induced by kainate in rats that developed convulsive SE to those who did not. Adult male Wistar rats were treated with kainic acid and tested behaviorally 5 months later. Rats that had experienced convulsive SE showed impaired performance on the spatial water maze and passive avoidance tasks, and on the context and tone retention tests following fear conditioning. In addition, they exhibited less anxiety-like behaviors than controls on the open-field and elevated plus-maze tests. Histologically, convulsive SE was associated with marked neuron loss in the hippocampal CA3 and CA1 fields, and in the dentate hilus. Rats that had not experienced convulsive SE after kainate treatment showed less severe, but significant impairments on the spatial water maze and passive avoidance tasks. These rats had fewer neurons than control rats in the dentate hilus, but not in the hippocampal CA3 and CA1 fields. Correlational analyses revealed significant relationships between spatial memory indices of rats and neuronal numbers in the dentate hilus and CA3 pyramidal field. These results show that a part of the animals that do not display intense behavioral seizures (convulsive SE) immediately after an epileptogenic treatment, later in life, they may still have noticeable structural and functional changes in the brain.

## Introduction

Temporal lobe epilepsy (TLE) can be associated with hippocampal cell loss (hippocampal sclerosis) and other structural changes in the medial temporal lobe region, such as fiber sprouting and synaptic reorganization [1], [2], [3]. It has also been repeatedly reported that TLE patients with hippocampal sclerosis may show learning and memory deficits, increased anxiety, depression and mood disorders [4], [5], [6]. Thus, it seems reasonable to assume that these behavioral changes are secondary to the TLE-related hippocampal neurodegeneration. However, it is also possible that they are not so straightforwardly related to classical hippocampal sclerosis, but may rather reflect some subtle functional or structural alterations in neuronal circuits, either associated with or even unrelated to epileptic seizures.

Most of the neuropathological aspects of TLE can be reproduced using appropriate animal models. For example, many studies have shown that the treatment of rats with kainic acid (KA), a specific agonist for glutamate receptors, induces acute status epilepticus (SE), accompanied by severe convulsive (motor) seizures, and that later in life these animals show spontaneous recurrent seizures, hippocampal cell loss and memory deficits [7], [8], [9]. However, in this epilepsy model some rats are considerably more resistant to KA-induced convulsive SE than the others and, for that reason, are usually excluded from the studies [9], [10], [11], [12], [13]. Thus, given that the research on the neuronal effects of KA in this subset of animals has been scarce [14], which represents an important gap in the understanding of epilepsy-related neuropathology, the present study was designed to directly compare the behavioral and neuroanatomical outcomes in rats that developed convulsive SE after kainate treatment to those that did not. We thought that the presence of behavioral deficits in KA-treated rats that had not experienced convulsive SE would support the hypothesis that at least some of the behavioral impairments seen in epilepsy can be explained by relatively mild structural or functional changes in the hippocampal region. To address this issue, we assessed the behavioral profile of KA-treated rats of both phenotypes using the Morris water maze [15], passive avoidance and fear conditioning tests, as well as in the open field and elevated plus maze tests. Additionally, absolute numbers of neurons in the dentate gyrus hilus and pyramidal layers of the hippocampus proper were estimated using stereological approaches in order to compare the hippocampal cell loss between the two groups.

## Materials and Methods

The handling and care of the animals were conducted according to the “Principles of laboratory animal care” (NIH publication No. 86-23, revised 1985) and Directive 2010/63/EU of the European Parliament and of the Council of 22 September 2010 on the protection of animals used for scientific purposes. The experimental protocol has been approved by the Ethics Committee of the Faculty of Medicine of Porto and the General Veterinary Direction (03.04.2012) for the FCT application grant PTDC/SAU-NSC/115506/2009. All efforts were made to minimize the number of animals used and their suffering.

### Animals and Treatments

Male Wistar rats, maintained under standard laboratory conditions, were used in this study. At 10 weeks of age (mean body weight 323±69 g), they were randomly divided into two groups: KA group (n = 28) and control group (n = 8). In the first group, rats were treated with KA according to the protocol developed by Dudek and coworkers and described in detail elsewhere [16], [17]. Briefly, the rats were injected every hour with 5 mg/kg of KA (i.p., Sigma) until the onset of convulsive SE, which was defined as the appearance of behavioral symptoms corresponding to stage 3, 4 or 5 seizures on the Racine scale [18], i.e. forelimb clonus, rearing, and rearing with falling. When necessary, treatment was continued in order to assure that SE lasted for at least 3 hours. All rats in this group demonstrated numerous wet-dog-shake seizures after either the second or third injection of KA. The SE, lasting 3–6 hours, was detected in 17 rats after 3–5 injections, with the mean cumulative dose of KA equal to 21±3 mg/kg. These rats were ascribed to the “behavioral status epilepticus” group (BSE; n = 17). Remaining 11 rats after 4 to 6 KA injections either became motionless (unchanged position in the observation cage for tens of minutes, weak reactivity to touching by experimenter’s hand), or showed excessive activity (long runs, jumping). Because we and others [17] have previously observed that continuation of the treatment of animals in these states often leads to their death, the treatment was terminated. The mean cumulative dose of KA received by these rats, which were ascribed to the “no behavioral status epilepticus” group (no-BSE; n = 11) was 24±4 mg/kg. It was previously reported that the treatment protocol employed in our study is associated with a relatively low mortality rate of the animals when compared to the single high-dose kainate treatments [16], [17]. However, because animal mortality is a prominent cause of bias in quantitative evaluation of neuronal loss [19], special efforts were made in order to further improve the survival rate in the kainate-treated rats. In particular, the animals were periodically injected with saline (s.c.) during the first 48 hours of the recovery period. On the following days, the rat diet was supplemented with apples that were sliced and left at the bottom of the cage. The rats that refused to eat or drink were hand-fed using a plastic syringe. Rats in the control group were injected with saline.

Following the treatments, all animals were given a 5-month recovery period. In the last week of each month, the behavior of rats in their home cages was videorecorded during the light phase of the 12-h light/dark cycle (between 08∶00 h and 20∶00 h) over a period of 5 consecutive days. The recording was performed using the multichannel video-tracking system EthoVision 8.5 (Noldus, The Netherlands). The video files generated by the system and stored on the hard disk of the computer were then analyzed in a fast-motion mode by a person blind to treatment groups using the VLC media player (VideoLAN, France). In addition, the rats were daily (except weekends) observed for spontaneous behavioral seizures during 1-h intervals between 09∶00 h and 10∶00 h, as well as during all the procedures associated with handling and behavioral testing.

### Open-field and Elevated Plus-maze Tests

Behavioral testing began when animals were 7 months old. Animals were handled 3 minutes per day during 5 days and subjected to the open-field test and plus-maze as previously described in detail [20]. The rats were counterbalanced so that, in each group, half received the open-field test first and the other half received the plus-maze test first. There was a 3-day interval between the two tests.

The behavior of rats in the open field (100×100×40 cm) was tested during 5-minute sessions. Distances traveled in the outer zone of the open field, defined as 20 cm from any wall, and in its inner zone were measured using the computerized EthoVision video-tracking system.

The elevated plus maze was arranged as a cross with two opposite open and two opposite closed arms (50×12 cm), connected by a common central square (12×12 cm). The rats were allowed to explore the apparatus for 5 minutes. Behavior of rats was recorded and analyzed using the video-tracking system. The percentages of time spent by rats in the open arms, closed arms, and in the central square were measured. The number of entries into the arms of the apparatus was also registered.

### Step-through Passive Avoidance and Fear Conditioning

Following a 3-day interval, all rats were submitted to the passive avoidance and fear conditioning tests. The rats were counterbalanced so that, in each group, half received the passive avoidance test first and the other half received the fear conditioning first. There was a 3-day interval between the two tests.

The apparatus used in the passive avoidance task was comprised of two adjacent compartments separated by a guillotine door [20]. The larger compartment (45×45×45 cm) was brightly lit and the smaller compartment (30×16×16 cm) was dark. The floor of both compartments was composed of stainless steel bars, 0.5 cm in diameter and spaced 1.2 cm apart, but only the floor of the dark compartment was wired to the stimulus generator (Hugo-Sachs Elektronik, Germany). On the first day, the rats were allowed to explore the apparatus with the guillotine door open for 5 minutes. The following day, each rat was placed into the brightly lit compartment and the latency to enter into the dark compartment was recorded. Upon entry into the dark compartment, the door was lowered and a 1-mA, 1-s footshock was delivered 3 times at 5-s intervals. Twenty four hours later, this procedure was repeated, with the exception that no footshock was delivered, and the latency to enter the dark compartment was again recorded.

All rats were given a single session of fear conditioning as previously described in detail [20]. The conditioning chamber (San Diego Instruments, USA) was equipped with a metal grid floor, wired to the stimulus generator and acoustic stimulus unit. The grid floor was composed of stainless steel bars, 0.6 cm in diameter, spaced 1.4 cm apart. The chamber was scented with 1% acetic acid solution (placed into base pan underneath the grid floor) in order to provide a distinct context odor. Rats were placed inside the apparatus and left undisturbed for 3 minutes. During next 3-minute period they received 5 tone-footshock conditioning trials, with 30-s intervals. Each conditioning trial consisted of a 10-s tone conditioned stimulus (80 dB, 2.8 kHz), which coterminated with a 1-s footshock unconditioned stimulus (0.8 mA) delivered via the grid floor.

One day later, all rats were submitted to retention tests. The order of testing was counterbalanced so that half of the rats in each group were tested for retention of the conditioned context by repeating the procedure employed during training (in the absence of the tone or footshock), whereas remaining rats were tested for retention of the conditioned tone in a different context. In the later test, the rats were placed into a novel chamber which was located in a novel behavioral room. The floor of the new chamber was composed of a piece of a black carpet. The chamber was scented with lemon instead of acetic acid. The animals remained in the chamber for a period of 6 minutes and the conditioned stimulus (10-s tone) was presented 5 times during the last 3 minutes of this period. Four hours later, these rats were tested for retention of the context as described above, whereas the animals from the context test were now subjected to the tone retention test.

All training and testing trials were recorded with a video camera connected to a SVHS recorder for subsequent analysis. The rat’s behavior was analyzed by an observer blinded with respect to experimental group. Freezing (defined as the absence of all movement other than that required for breathing) was scored if the rat remained inactive for at least 3 seconds. The percentage of accumulated time spent freezing was calculated.

### Morris Water Maze Test

The procedure of the water maze training, previously described in detail [21], initiated one week following the termination of the passive avoidance and fear conditioning tests. The maze consisted of a black circular tank, 180 cm in diameter and 50 cm deep, and was located in a corner of a room containing extramaze cues. The apparatus was filled with water at room temperature (21±1°C) to a depth of approximately 35 cm. The water was made opaque by adding a non-toxic paint. The maze was divided, by imaginary lines, into four equal-size quadrants. An escape platform, 10 cm in diameter, was placed in the center of one of the quadrants. It was located 2 cm below the surface of the water. The animals were trained to find the escape platform during 14 consecutive days with two 60-s daily trials separated by a 30-s inter-trial interval. Each rat was placed in the water facing the pool wall at one of the four starting points that were used in a pseudo-random order so that each position was used once in each block of four trials. However, in each trial the starting positions were the same for all animals. If the rats did not find the escape platform within 60 s, the experimenter guided them to the platform where they were allowed to remain for 15 s. The platform location was not changed during the 14-day training period. The swim path length and swim speed were registered using the EthoVision video-tracking system. One day after completion of the acquisition, animals were submitted to a single 60-s probe trial in which the platform was removed from the pool. The number of times the rats swam through the zone where the platform had been located (platform crossings) and the percentage of time spent by rats swimming in the training quadrant were recorded.

Performance of animals on the visible platform task was assessed during a 2-day period. The rats were given 1 block of 4 trials per day separated by 30-s inter-trial intervals. The platform, painted in white, was exposed 3 cm above the water surface. The position of the platform was different in each trial. The distances swum to locate the platform were recorded and averaged across 8 trials.

### Tissue Preparation

Following the completion of the behavioral experiments, six animals in each group, selected at random, were deeply anesthetized with pentobarbital (90 mg/kg) and injected intracardially with 0.1 mL of a heparin solution, followed by 1 mL of 1% sodium nitrite in saline. Then, they were perfused transcardially with 150 mL of 0.1 M phosphate buffer (pH 7.4) for vascular rinse, followed by 250 mL of a fixative solution containing 4% paraformaldehyde in phosphate buffer. The brains were removed from the skulls, immersed for 2 hours in the fixative, and infiltrated during 36 h in 10% sucrose solution at 4°C. After the frontal poles were trimmed away, the blocks were mounted on a vibratome and sectioned in the coronal plane at 40 µm. Every sixth section containing the hippocampal formation was collected using a systematic random sampling procedure [22], which provided an average of 14 sections per animal, mounted serially on gelatin-coated slides and air-dried. The sections were stained with a Giemsa, dehydrated in a series of ethanol solutions (50%, 70%, 90% and 100%) and coverslipped using Histomount (National Diagnostics, Atlanta, GA, USA).

### Morphological Analysis

The Giemsa-stained tissue sections were visualized and imaged using an Axio Scope.A1 microscope equipped with a AxioCam MRc5 digital camera (Zeiss, Germany). The total numbers of neurons were estimated by applying the optical fractionator method [23]. The boundaries of the hilus of the dentate gyrus and of the CA3 and CA1 hippocampal fields were consistently defined at all levels along the rostrocaudal axis of the brain on the basis of cell morphology and cytoarchitectonic criteria [24]. Neurons belonging to the CA2 hippocampal field were included in the CA3 region. Estimations were carried out using the C.A.S.T.-Grid System (Olympus, Denmark). Beginning at a random starting position, visual fields were systematically sampled along the x and y axes, using a raster pattern procedure. Neurons were counted in every frame using the optical disector at a final magnification of ×2,000. Tissue thickness was estimated at each counting frame and guard zones of 2 µm were implemented. The nucleus of the neurons was used as the counting unit. The coefficient of error (CE) of the individual estimates was calculated according to Gundersen et al. [25] and it ranged between 0.06 and 0.09.

### Statistical Analysis

Before conducting statistical comparisons, data were tested for normality using the Shapiro-Wilk’s W test, which is known to be the most powerful normality test, especially in the case of small sample sizes [26]. Because all data samples passed the normality test (*p*>0.05), they were analyzed for statistical significance using parametric tests. Data derived from the passive avoidance test, acquisition trials of the water maze task, and training trials and tone retention trials of the fear conditioning test were analyzed using repeated measures ANOVA. The remaining data were analyzed using one-way ANOVA. Newman-Keuls post hoc test was used where appropriate. Because some of the behavioral tests were presented in a counterbalanced manner, the data derived from these tests were collapsed across order of presentation. Pearson’s product moment regression model was used to analyze correlations between the neuron numbers and the two behavioral indices derived from the water maze test: the percentage of savings assessed by the difference in the mean distance swum between the first four trials and the last four trials (index of learning), and the percentage of time spent by rats swimming in the quadrant where the platform had been located during training (index of retention). All behavioral data are presented as the mean±SEM, while morphological results are expressed as the mean±SD. Differences were considered as significant at the *p*<0.05 level.

## Results

### Monitoring during the Recovery Period

Despite all the efforts made to reduce the mortality rate in KA-treated rats (see above), a significant proportion of them have died during the recovery period. In particular, from the 17 rats that had experienced convulsive SE 3 rats have died within 48 h following the treatment and 3 rats have died during the first month of the recovery period. From the 11 rats that did not show motor manifestations of SE 2 rats have died within 48 h following the treatment. Repeated behavioral seizures of stage 3 to 5 on the Racine scale were observed in a total of 9 rats: by the end of the first month of recovery – in just 1 rat; by the end of the second and third months – additionally in 3 and 5 rats, respectively. Interestingly, during the treatment with KA, all of these rats showed a typical pattern of behavioral SE, that is, continuous stage 3 to 5 seizures on the Racine scale. Three of the rats in this group had motor seizure attacks during the behavioral tests: 1 rat – during the water maze training (twice) and 2 rats during the tone test of the fear conditioning procedure. The respective trials were repeated for these animals on the next day. None of the rats had motor seizures during the 1-h intervals preceding or following the behavioral tests. The remaining 2 rats, which experienced convulsive SE during the treatment with KA, showed no spontaneous stage 3, 4 or 5 seizures during the entire 6-month observation period (including the period of behavioral tests). However, one of the experimenters detected in these 2 rats repeated mouth and facial movements (stage 1 seizures on the Racine scale), but this observation was not confirmed by other experimenters. Thus, the final group size for BSE rats was n = 11. No signs of behavioral seizures (stages 1–5 on the Racine scale) were detected in the remaining 9 KA-treated rats. These rats, in contrast, did not show clearly expressed motor signs of SE during the treatment with KA (no-BSE group; final n = 9). No behavioral abnormalities were observed in the control group.

### Behavioral Effects of KA Treatment in the Open-field and Elevated Plus-maze

**Figure 1:**
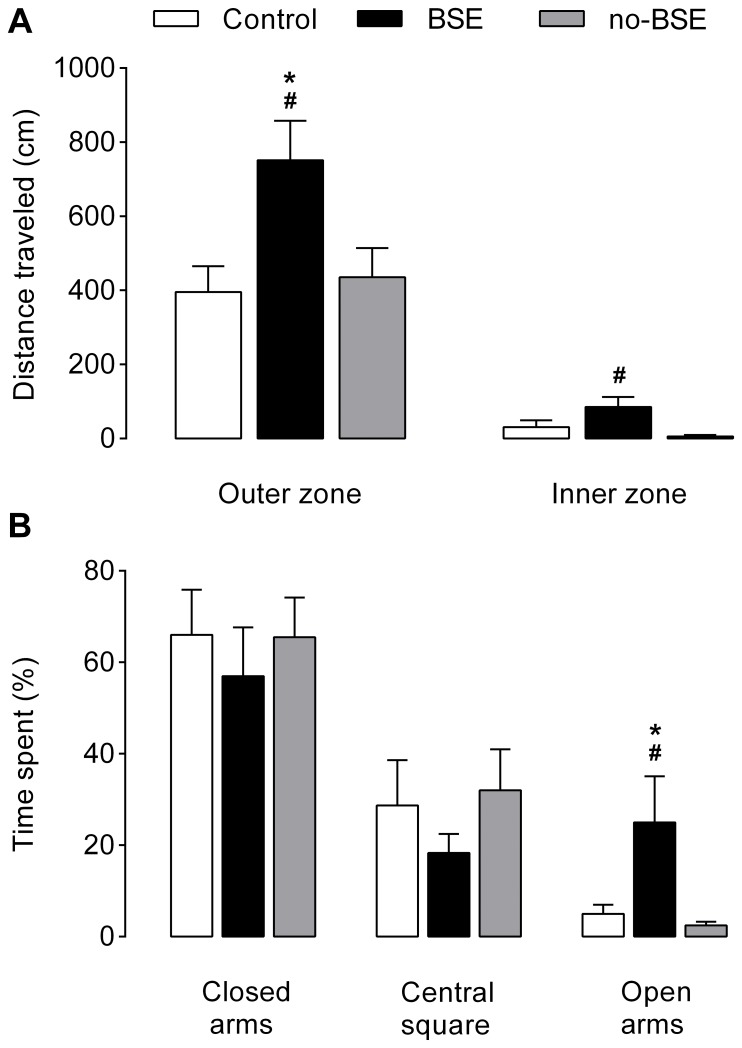
Behavior of kainate-treated rats in the open-field (A) and elevated plus-maze (B) tests. The y-axis represents the distances travelled by rats during the 5-min testing sessions in the outer and inner zones of the open-field apparatus, and in the central zone and open and closed arms of the plus-maze apparatus. Note that rats who had experienced behavioral status epilepticus (BSE group) traveled longer distances in the outer and inner zones of the open field when compared to control rats and rats that had not experienced behavioral status epilepticus (no-BSE group), respectively. In the plus-maze test, rats in the BSE group spent more time in the open arms of the apparatus than rats in the other two groups. *p<0.05 vs. control group and ^#^ *p*<0.05 vs. no-BSE group. Data are presented as the mean±SEM.

The results obtained in the open-field tests are shown in Fig. 1A. ANOVA revealed a significant effect of the treatment on the locomotion activity of rats both in the outer zone of the apparatus (*F*_2,25_ = 4.70, *p*<0.05) and in its inner zone (*F*_2,25_ = 4.07, *p*<0.05). Post-hoc comparisons showed that animals from BSE group traveled significantly longer distances in the peripheral zone of the open field than did control rats and no-BSE rats (*p*<0.05). Similarly, rats from BSE group showed increased locomotion in the inner part of the open field. However, in this zone, the difference was significant only when BSE group was compared to no-BSE group (*p*<0.05), but it did not reach the level of significance when compared to control group (*p* = 0.08).

Rats in all groups showed clear preference to protected areas of the plus maze, as they spent more time in the closed arms and central zone of the apparatus in comparison to its open arms (Fig. 1B). However, although the percentages of time spent by rats in the closed arms and central zone did not differ between the groups (*Fs*<1, *p*>0.05), ANOVA revealed a significant effect of the treatment on the time spent by rats in the open arms (*F*_2,25_ = 3.31, *p*<0.05). Particularly, rats in the BSE group spent significantly more time in the open arms than those from control group and no-BSE group (*p*<0.05). No significant differences were detected between the groups regarding the total number of entries into the arms of the apparatus.

### Effects of KA Treatment on Passive Avoidance and Conditioned Freezing Behavior

**Figure 2:**
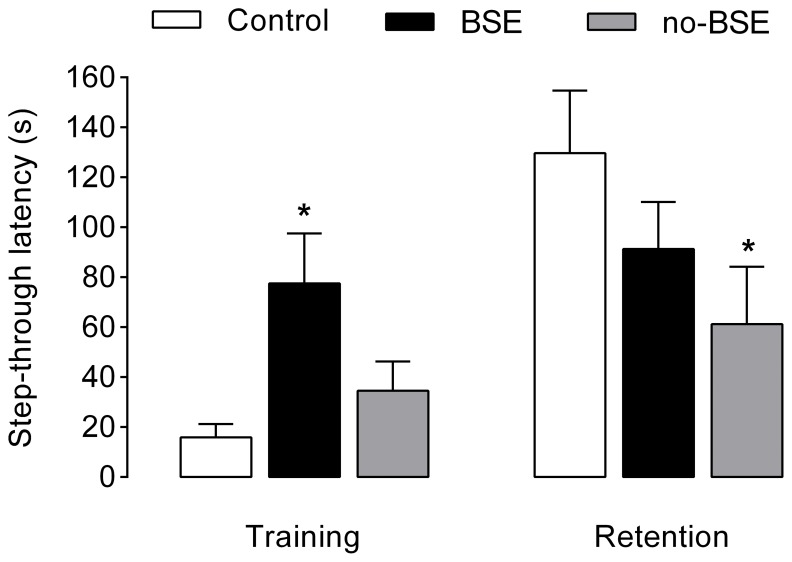
Performance of kainate-treated rats on the passive avoidance test. The y-axis represents the mean (±SEM) step-through latencies during training trial as well as on the retention trial given 24 h later. Note that rats which did not show behavioral signs of status epilepticus following the treatment with kainate (no-BSE group) showed lower retention scores than did control rats. In contrast, rats that had experienced behavioral status epilepticus (BSE group) showed increased baseline entry latency, but appeared normal on the retention test. *p<0.05 vs. control group.

The results of the passive avoidance testing are shown in Fig. 2. Repeated measures ANOVA did not reveal a significant main effect of treatment on the performance of rats on this task (*F*_2,25_ = 1.47, *P* = 0.25). However, there was a significant effect of training (*F*_1,25_ = 14.30, *p*<0.001) and a significant interaction between these two effects (*F*_2,25_ = 5.09, *p*<0.01). Post-hoc comparisons showed that, although rats in no-BSE group did not differ from control rats in the baseline latency to enter the dark compartment, on the retention trial, the entry latencies were significantly reduced in this group relative to controls (*p*<0.05). The latencies to enter the dark compartment on the retention test were not significantly different between control group and BSE group. However, the baseline latency was significantly increased in BSE rats relative to control rats (*p*<0.05), suggesting that the high entry latencies registered in this group on the retention test might be related to a decreased motivation to enter the dark compartment of the apparatus, for example, due to reduced levels of anxiety.

**Figure 3:**
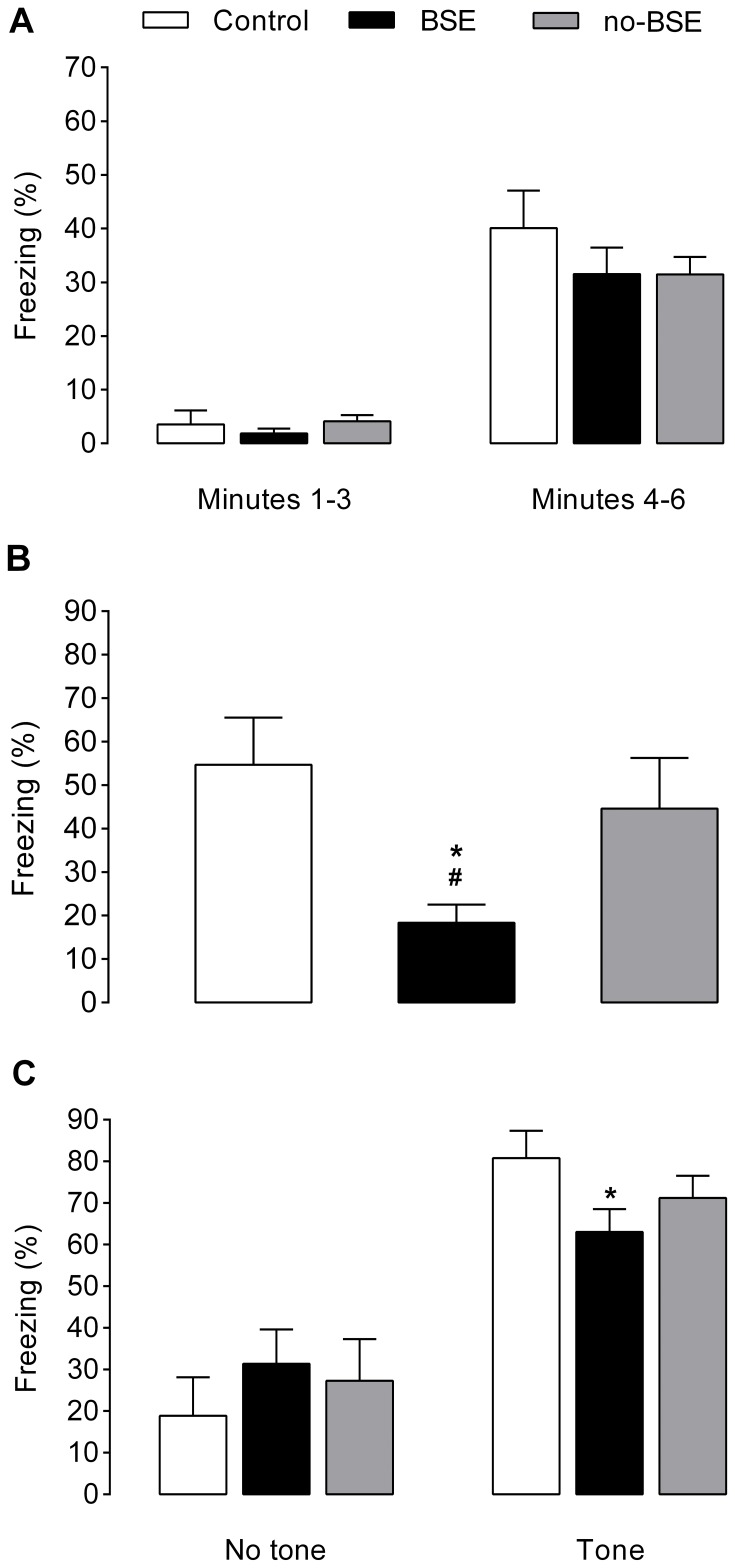
Effects of kainate treatment on the acquisition and retention of conditioned fear. The y-axes represent percentage of freezing time for each of the 3-minute periods of the acquisition session (**A**), during the context retention test (**B**), and during each of the 3-minute periods of the tone retention test which was performed in a novel context (**C**). No tone or footshock was delivered during the first 3-minute periods of the acquisition session (**A**) and of the tone retention test (**C**). Note that rats that had experienced behavioral status epilepticus (BSE group) showed reduced levels of fear, freezing significantly less than control rats on both retention tests. However, no-BSE group (composed of rats that had not experienced behavioral status epilepticus after kainate treatment) and control group did not differ significantly on these tests. *p<0.05 vs. control group and ^#^ *p*<0.05 vs. no-SE group. Data are presented as the mean±SEM.

Conditioning produced an increase in the amount of freezing time in either group (*F*_1,25_ = 156.11, *p*<0.0001; Fig. 3A) and examination of the data revealed no significant differences between the groups. Once placed into the training chamber 1 day later, the animals from all groups displayed increased levels of freezing behavior (Fig. 3B). However, the amount of freezing differed between the groups (*F*_2,25_ = 4.68, *p*<0.05), so that rats in the BSE group froze to the familiar context significantly less than control rats and no-BSE rats (*p*<0.05). However, no-BSE and control groups did not differ significantly in this test. In the novel context, the levels of freezing displayed by all groups in the absence of the tone were inferior to those observed during the tone presentation (*F*_1,25_ = 99.42, *p*<0.0001; Fig. 3C). Repeated-measures ANOVA of these data did not reveal a significant main effect of treatment, but yielded a significant treatment by tone presentation interaction (*F*_2,25_ = 3.29, *p*<0.05). Post-hoc tests showed that, although in the absence of the tone the 3 groups did not differ with respect to the amount of freezing, during the tone presentation, the rats in BSE group showed relatively low levels of fear, freezing significantly less than control rats (*p*<0.05).

### Water Maze Navigation

**Figure 4:**
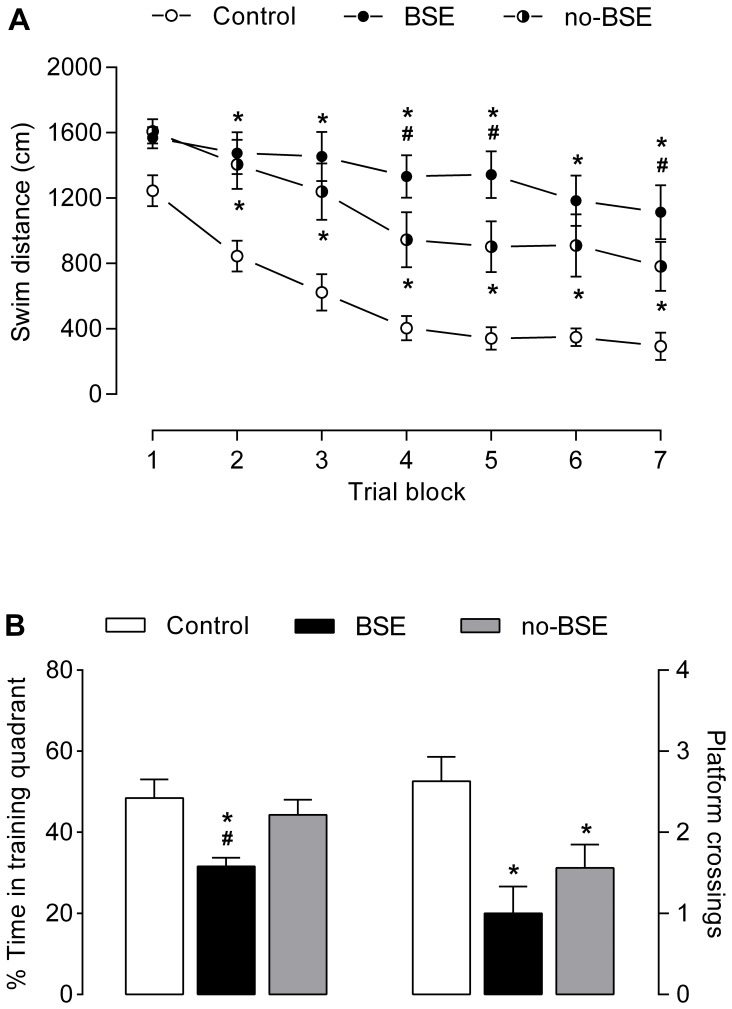
Effects of kainate treatment on the performance of rats on the water maze. (**A**) The y-axis represents the mean swim distance (±SEM) in centimeters to reach a hidden platform throughout the acquisition of the water maze task. The data were averaged across seven blocks of four consecutive trials each (the x-axis). Note that the performance of rats that had experienced behavioral status epilepticus (BSE group) on this task was extremely poor. However, spatial learning of the rats that showed no convulsive status epilepticus (no-BSE group) was also considerably impaired. *p<0.0001 for BSE group vs. control group and *p*<0.001 for no-BSE group vs. control group; ^#^ *p*<0.05 vs. no-BSE group. (**B**) The y-axes represent the percentage of time spent by rats in the training quadrant of the water maze during the probe trial and the number of times they swam through the zone where the platform had been located (platform crossings). Unlike control rats, rats in the BSE group did not show a preference for the training quadrant of the water maze and were impaired in their ability to localize the former platform position. However, the acuity of spatial search was also impaired in the no-BSE rats, as indicated by the fact that they crossed the former platform position less frequently than did control rats. *p<0.05 for no-BSE group vs. control group and *p*<0.01 for BSE group vs. control group; ^#^ *p*<0.05 vs. no-BSE group. Data are presented as the mean±SEM.

The mean distances swum by rats in the control, BSE and no-BSE groups to locate the hidden platform in the place learning task are presented in Fig. 4A. The statistical analysis by repeated measures ANOVA showed that the rats included in this experiment progressively improved their ability to find the platform over the 14 days of acquisition (*F*_6,150_ = 25.980 *p*<0.00001). However, the overall performance of the rats on this task differed among the groups studied, as indicated by a significant main effect of treatment (*F*_2,25_ = 12.89, *p*<0.0001) and significant treatment × trial block interaction (*F*_12,150_ = 1.86, *p*<0.05). Post-hoc tests for the main effect of treatment revealed that animals from the BSE group and no-BSE group performed the task less well than did control animals (*p*<0.001 and *p*<0.01, respectively), but animals in the BSE group were more severely impaired than those in the no-BSE group (*p*<0.05). Post-hoc tests for the treatment × trial block interaction showed that BSE group performed significantly worse than control group on the 2^nd^ to 7^th^ trial blocks (*p*<0.0001). In turn, rats in the no-BSE group also traveled greater distances than did control rats on the 2^nd^ to 7^th^ trial blocks (*p*<0.001). Moreover, rats from the BSE group were less efficient in finding the escape platform than rats from the no-BSE group on the 4^th^, 5^th^, and 7^th^ trial blocks (*p*<0.05).

Behavioral measures obtained on the probe trial are shown in Fig. 4B. ANOVA revealed a significant effect of treatment on the total percentages of time spent by rats swimming in the training quadrant (*F*_2,25_ = 7.01, *p*<0.01) and a significant effect of treatment on the number of times they crossed the former location of the escape platform (platform crossings; *F*_2,25_ = 6.40, *p*<0.01). Post-hoc tests showed that both control and no-BSE rats spent more time in the training quadrant than did rats in the BSE group (*p*<0.01 and *p*<0.05, respectively). However, the no-BSE group did not differ from the control group on this measure, suggesting that rats in both these groups, unlike those in the BSE group, were able to learn a spatial strategy when searching for the escape platform. Nevertheless, the acuity of spatial search was impaired in both KA-treated groups, as indicated by the fact that control rats crossed the former platform position more frequently when compared to both BSE group (*p*<0.01) and no-BSE group (*p*<0.05).

ANOVA revealed that there was a significant main effect of treatment on the speed of swimming averaged across all trials of the water maze acquisition (*F*_2,25_ = 3.29, *p*<0.05). The animals from the BSE and no-BSE groups swam somewhat faster than control rats (24.7±1.2 and 22.3±1.5 cm/s vs. 20.1±1.1 cm/s, respectively). However, only BSE rats differed significantly from control rats on this measure (*p*<0.05). Animals in all groups were able to learn the visible platform task. The distances swum to locate the platform, averaged over eight trials, were 398±102 cm in the control group, 513±115 cm in the BSE group and 466±95 cm in the no-BSE group. ANOVA failed to reveal a significant effect of treatment on this parameter. These data indicate that, with respect to sensorimotor functions, KA-treated rats were not impaired when compared to control rats.

### Effects of KA Treatment on Hippocampal Morphology

**Figure 5:**
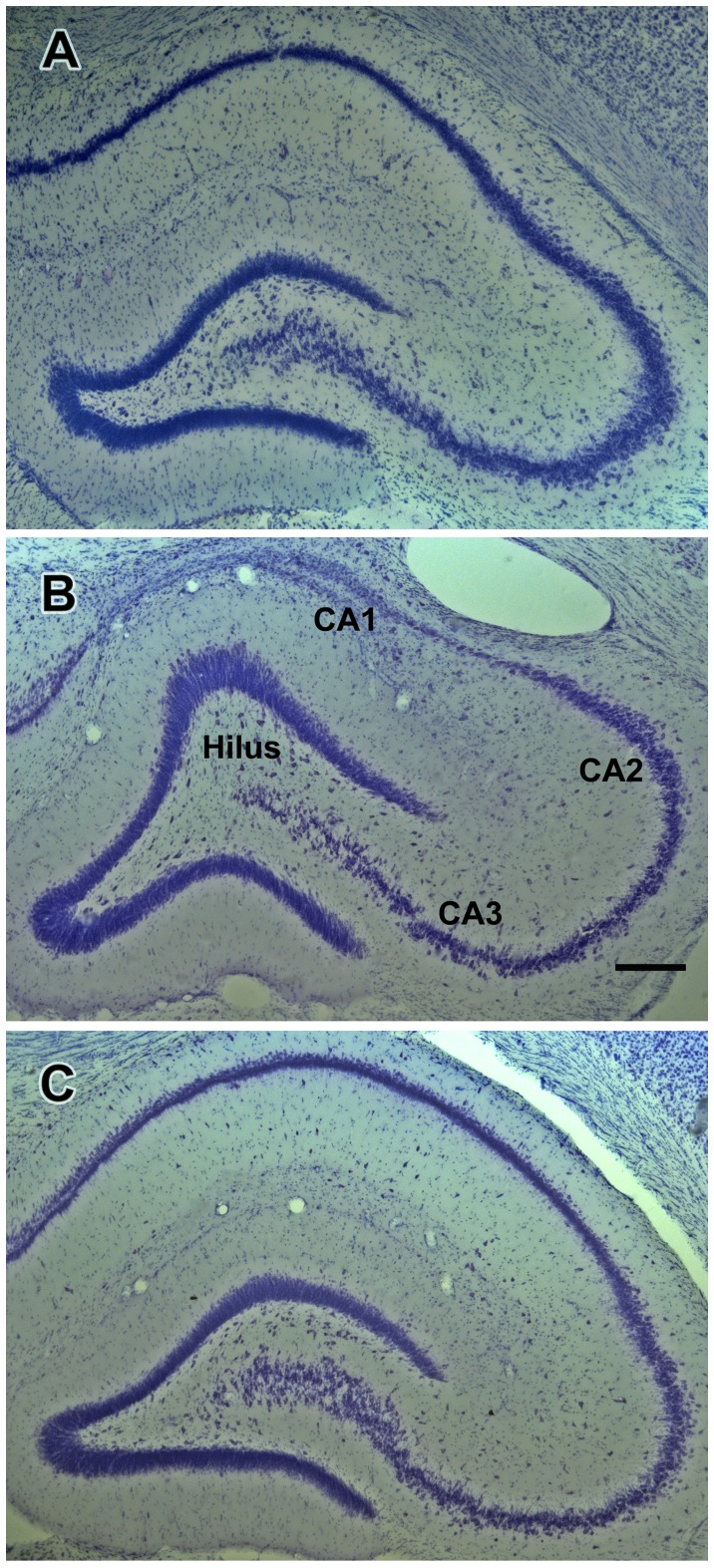
Effects of kainate treatment on hippocampal morphology. Photomicrographs of representative Nissl-stained coronal sections containing the dentate gyrus, CA3 and CA1 hippocampal fields taken from a control rat (**A**), from a rat in the behavioral status epilepticus (BSE) group (**B**) and from a kainate-treated rat that had not experienced behavioral status epilepticus (no-BSE; **C**). Note that the density of neurons in the dentate hilus is considerably reduced in the rats with (**B**) and without (**C**) behavioral SE when compared to the control rat (**A**). Note also dramatic loss of the CA3 and especially CA1 neurons in the rat from the BSE group; neurons in the CA2 field are relatively preserved (**B**). Scale bar shown in (**B**) = 200 µm.

**Figure 6:**
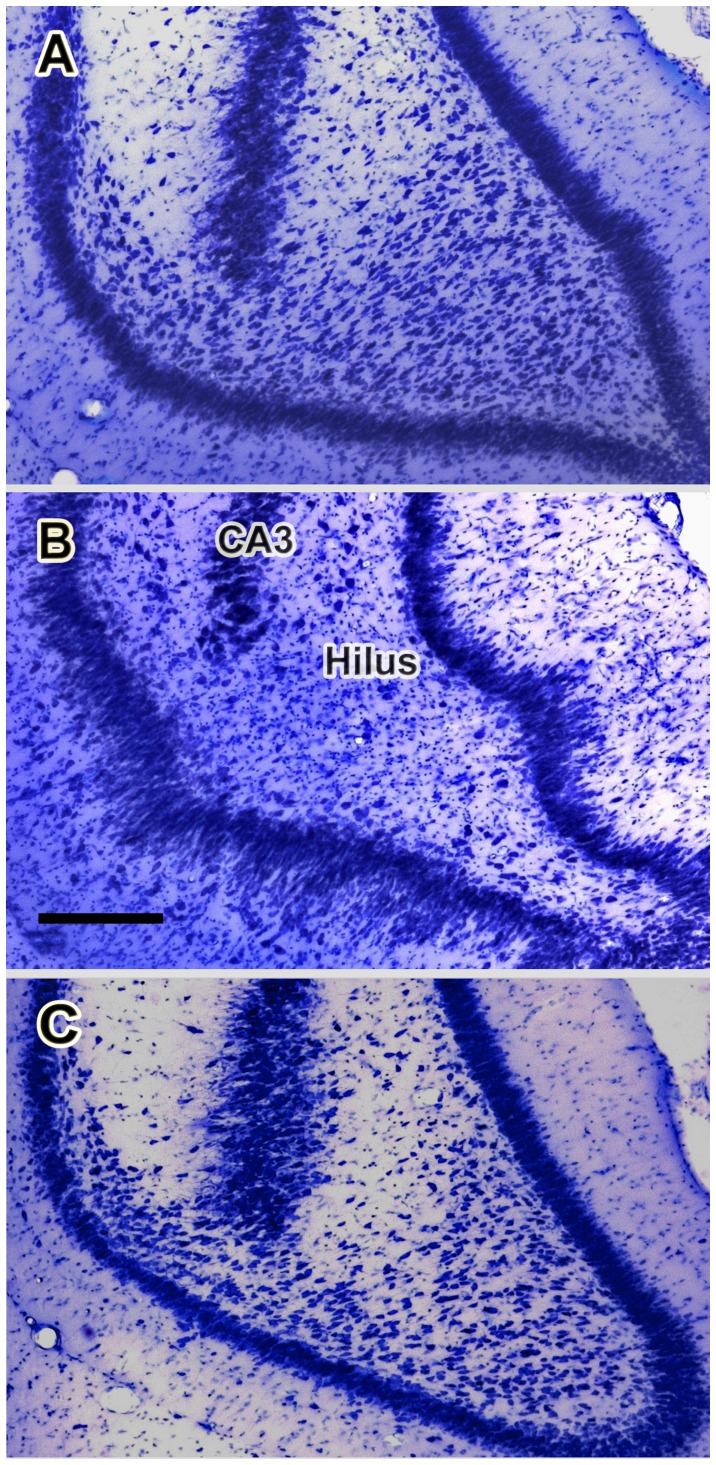
Kainate-induced loss of neurons in the ventral dentate hilus. Photomicrographs of representative Nissl-stained coronal sections containing the ventral portion of the dentate gyrus taken from a control rat (**A**), from a rat in the behavioral status epilepticus (BSE) group (**B**) and from a kainate-treated rat that had not experienced behavioral status epilepticus (no-BSE; **C**). Note that the density of neurons in the dentate hilus is dramatically reduced in the rat that experienced behavioral SE (**B**). In striking contrast, most of the neurons of the ventral hilus are preserved in the rat from the no-BSE group (**C**). Scale bar shown in (**B**) = 250 µm.

Representative photographs of brain sections illustrating treatment-related morphological changes in the hippocampal formation of rats in the BSE and no-BSE groups are shown in Fig. 5 and Fig 6. The qualitative observation of the sections inferred that the neuronal density of the CA3 and CA1 pyramidal cells was considerably smaller in BSE-rats than in control rats and no-BSE rats. In some hippocampal regions, CA1 pyramids were practically absent in BSE rats (Fig. 5B). In addition, the dentate gyrus was deformed and its granule cell layer was dispersed in animals that experienced behavioral SE. Consistent with prior anatomical studies [27], [28], the density of hilar neurons in control rats was higher in the sections obtained from the temporal (ventral) pole of the dentate gyrus when compared to those obtained from its septal (dorsal) pole (Fig. 5A and Fig. 6A). The density of hilar neurons was reduced in KA-treated rats that experienced convulsive SE throughout the entire dentate gyrus, but neuron loss was particularly extensive in the ventral hilus (Fig. 6B). However, in rats from the no-BSE group hilar neuron loss was less extensive and most of the neurons of the ventral hilus were preserved (Fig. 6C).

**Figure 7:**
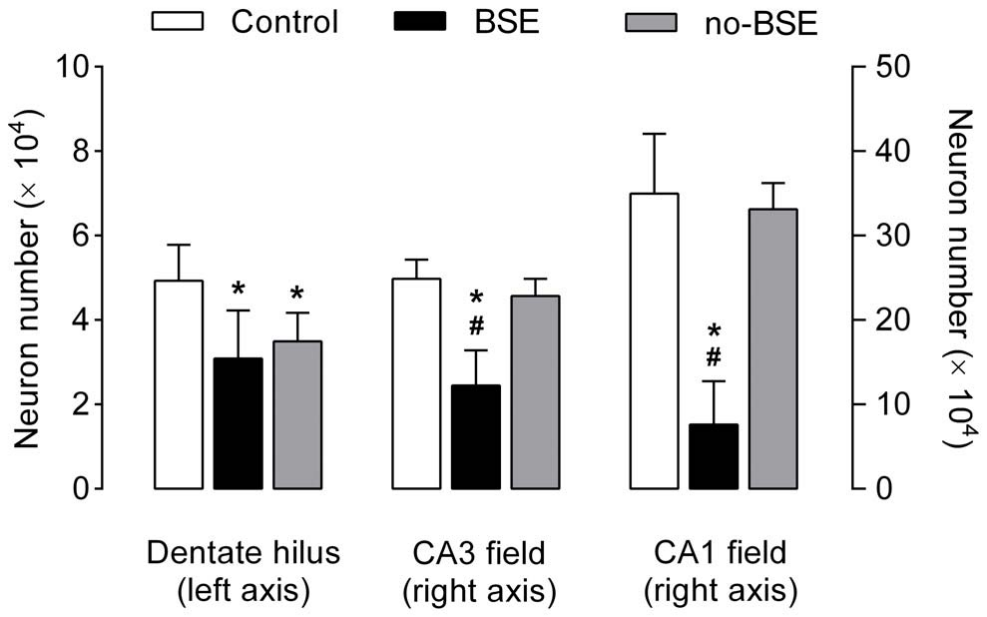
Loss of hippocampal neurons in kainate-treated rats. The y-axes represent the total numbers of neurons (mean±SD) in the dentate hilus and hippocampal CA3 and CA1 pyramidal fields in control rats and in kainate-treated rats with and without behavioral status epilepticus (BSE group and no-BSE group, respectively). The number of hilar neurons is reduced in both groups of kainate-treated rats when compared to control group. Note that the number of cells in the CA3 and CA1 pyramidal regions was strikingly reduced in rats from the BSE group, but not in those from the no-BSE group. Dentate hilus: *p<0.05 for no-BSE group vs. control group and *p*<0.01 for BSE group vs. control group; CA3 and CA1 fields: *p<0.001 vs. control group and ^#^ *p*<0.001 vs. no-BSE group.

The estimates of the total numbers of neurons in the hilus of the dentate gyrus, and in the CA3 and CA1 fields of the hippocampus proper are shown in Fig. 7. Analysis of these data revealed that there was a significant main effect of seizures on neuron numbers in all three hippocampal subdivisions: dentate hilus (*F*_2,15_ = 6.77, *p*<0.01), pyramidal CA3 field (*F*_2,15_ = 31.13, *p*<0.0001) and pyramidal CA1 field (*F*_2,15_ = 30.72, *p*<0.0001). Post-hoc comparisons showed that in rats in which KA treatment resulted in the development of behavioral SE there was a significant neuron loss in the dentate hilus (40%; *p*<0.01), and hippocampal CA3 (50%; *p*<0.001) and CA1 (80%; *p*<0.001) fields. In sharp contrast, in rats from the no-BSE group there was a significant neuron loss only in the dentate hilus (30%; *p*<0.05), but not in the hippocampal pyramidal fields.

### Relationship between KA-induced Neuron Loss and Spatial Learning and Memory

**Figure 8:**
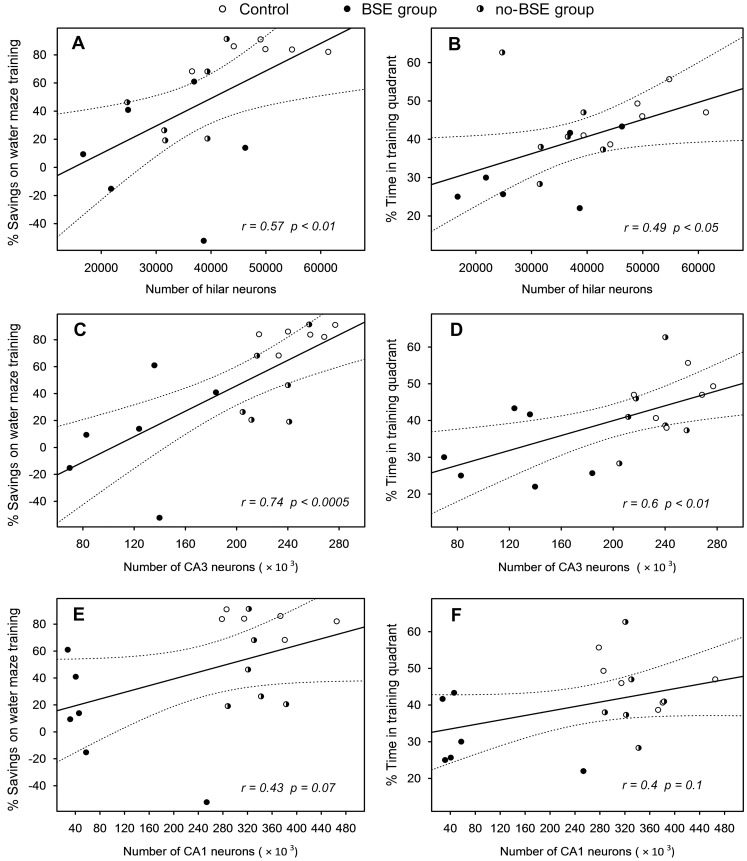
Correlations between hippocampal neuron numbers and spatial learning and memory indices. The x-axes represent the total numbers of neurons in the dentate hilus (**A**, **B**), and hippocampal CA3 (**C**, **D**) and CA1 (**E**, **F**) pyramidal fields in control and kainate-treated rats. The y-axes represent the percentages of savings during water maze training, i.e. the differences, in %, between the mean distances swum by rats on the first four trials and on the last four trials (**A**, **C**, **E**), and the percentages of time spent by rats in the training quadrant of the water maze during the probe trial (**B**, **D**, **F**). Data obtained from 6 animals randomly selected from each group (final n = 18) were used in the correlational analyses. Correlation coefficients are shown at the bottom of each plot and dashed lines represent 95% confidence intervals.

Correlational analyses were conducted to determine whether individual variations in learning and memory abilities of rats are related to KA-induced loss of hippocampal neurons. The analyses were conducted separately for hilar neurons, and CA3 and CA1 hippocampal pyramidal neurons. Data obtained from 6 animals from each group, whose brains were randomly selected for histological assessment, were included in the analysis (final n = 18). The results of the correlational analyses, shown in Fig. 8, revealed significant correlations between the total number of hilar neurons and two behavioral indexes of spatial learning and memory, i.e. the percentage of savings between the first four trials and the last four trials of the water maze acquisition (*r* = 0.57, *p*<0.01; Fig. 8A) and the percentage of time spent in the training quadrant during the probe trial (*r* = 0.49, *p*<0.05; Fig. 8B). Interestingly, both behavioral measures tend to exhibit even stronger correlations with the total number of neurons in the CA3 hippocampal field (*r* = 0.74, *p*<0.0005 and *r* = 0.6, *p*<0.01 for the percentage of savings and percentage of time in the training quadrant, respectively; Fig. 8C, D). In contrast, we found no significant correlations between the two behavioral indexes and the total number of the CA1 pyramidal neurons (Fig. 8E, F).

## Discussion

The results of this experiment show that rats treated with similar doses of KA may have two quite distinct sets of brain changes. In particular, the neuropathological sequelae of the treatment were different in the following ways: (1) in one group of rats KA induced a clear pattern of convulsive SE including repeated stage 4 and 5 seizures on the Racine scale, but this was not observed in remaining rats; (2) majority of the rats that had experienced behavioral SE developed spontaneous motor seizures 1 to 3 months later, whereas the rats in the other group did not; (3) histological analysis revealed massive loss of hippocampal pyramidal neurons in rats that had experienced behavioral SE, whereas no pyramidal cell loss was detected in rats who did not show motor signs of SE; in either group of KA-treated rats, the total number of neurons in the hilus of the dentate gyrus was reduced; (4) although rats in both KA-treated groups were impaired in behavioral tests, the impairments were significantly more profound and wide-ranging in the BSE group. These data are consistent with previous studies showing that animals of the same strain may have different seizure susceptibility [14], [29], [30], [31], which is believed to have both genetic [29], [30] and environmental [32] underpinnings.

Rats in the BSE group were not only impaired in learning the precise platform position in the water maze, but were, in fact, unable to adopt a spatial strategy to solve the task. In addition, they showed severely impaired performance on the passive avoidance task and on the fear conditioning tests. Rats in this group were hyperactive in the open-field test and spent more time walking on the open arms of the elevated plus maze, suggesting a deficient emotional responsiveness. Our results are consistent with many prior studies in which behavioral changes were detected in animals following SE induced by either electrical stimulation [11], [33] or injections of chemoconvulsants (pilocarpine [34], [35], lithium-pilocarpine [9], [13], [36], and kainate [8], [9], [11], [12], [13], [37], [38] models). However, the behavioral deficits observed in the present study appear to be somewhat more pronounced than those reported in prior studies using the same model of SE [9], [39], [40]. This discrepancy is likely due to differences in experimental conditions between different studies. For example, it has been reported that the induction of SE in adult rats is followed by more robust behavioral deficits than in premature rats [8], [37], [38] and that old rats, in turn, are more susceptible to SE when compared to adult rats [12]. In addition, it has been shown that the seizure-related behavioral impairments are strain-specific [9], [13], [31], [41]. Furthermore, it is also well documented that different treatment protocols, e.g. duration of SE, may be associated with very different neuropathological outcomes in experimental animals [9], [16], [42]. It may be also noted that we performed behavioral testing of rats approximately 5 months after the induction of SE, that is, at a time when seizure frequency is maximal [43]. For comparison, studies on animals just 2 or 3 months post-SE, when seizures are relatively less frequent and neuroanatomical changes are likely to be less dramatic, generally reveal less severe behavioral deficits [9], [40]. However, it remains to be seen whether SE-related behavioral deficits actually worsen with increased duration of epileptic state. In fact, it has been recently reported that animals tested two months and twenty months after the induction of SE showed similar cognitive impairments [44].

There are a few prior studies in which animals that did not develop convulsive SE after the respective treatments were evaluated behaviorally in parallel to those who did. In particular, Szyndler et al. [36] reported that lithium-pilocarpine-treated rats resistant to develop SE did not differ behaviorally from control rats, whereas rats that developed SE were impaired in the fear conditioning and open-field tests. Müller et al. [34] have shown that pilocarpine-treated mice without motor signs of SE differed significantly from control mice in several measures derived from the open-field, hole-board and light-dark transition tests (mainly related to their increased activity), but were intact, unlike mice which had behavioral SE, on the elevated plus-maze, novel object exploration and spatial water maze tests. However, histological analysis carried out in the latter study revealed significant loss of hippocampal neurons only in mice that developed convulsive SE [34]. Interestingly, it has been also reported that treating rats with KA during early postnatal period induces electrographic and mild behavioral seizures, but does not evoke a typical pattern of SE, i.e. stage 4–5 seizures on the Racine scale [37], [38]. Yet, later in life, these rats show significant cognitive deficits accompanied by long-term impairments in hippocampal plasticity [37], [38]. In this respect, it is worth noting the results of a recent study by Suárez et al. [14] who reported that treating rats with KA, even if it induces neither motor SE, nor spontaneous seizures later in life, may lead to enduring changes in synaptic plasticity. In the present study, KA-treated rats that did not develop motor SE had cell loss in only one of the hippocampal subdivisions, the dentate hilus, but were impaired on the passive avoidance and spatial water maze tasks. Thus, taken together, these findings indicate that a part of the animals that do not manifest intense behavioral seizures immediately after an epileptogenic treatment, later in life, they may still have conspicuous structural and functional changes in the brain.

It is well documented that epilepsy models based on the induction of SE are typically associated with marked neuronal loss in various brain regions, such as the hippocampal formation, amygdala, thalamus, piriform, entorhinal, perirhinal, retrosplenial and olfactory cortices [7], [9], [10], [11], [33], [35], [45], [46], [47], many of which are implicated in cognitive and emotional processes [15], [48], [49], [50], [51], [52]. Therefore, it is likely that brain damage induced by SE and subsequent spontaneous seizures is directly responsible for the behavioral deficits observed in these models. In effect, it has been recently shown that hippocampal volume of epileptic rats significantly correlates with their learning and memory abilities [9], [53]. Similar relationships were found between the volumetric estimates of the amygdala and the indices of emotionality [9],[53]. In this study, we detected marked loss of hippocampal CA3 and CA1 pyramidal neurons in rats that had experienced behavioral SE, whereas in rats without behavioral manifestations of SE these neuronal populations were preserved. However, the loss of hilar neurons was detected in both KA-treated groups, even though it was predictably greater in BSE group than in no-BSE group. Thus, our data suggest that hilar cell loss alone may be sufficient to cause functional deficits in hippocampal circuits as indicated by the impaired performance of no-BSE rats on the water maze and passive avoidance tests. This suggestion is consistent with the evidence that hilar neurons may influence mnemonic processes by synchronizing the activity of the dentate gyrus granule cells and, more generally, by modulating the transmission of information from the entorhinal cortex to the hippocampal pyramidal fields [54], [55]. This possibility is also supported by the results of the correlational analyses which revealed significant correlations between individual spatial memory indices of rats used in this experiment and the total number of hilar neurons, but not of CA1 pyramidal neurons. The strongest correlation, however, was found between the cognitive indices and the total number of CA3 neurons. This result may appear surprising given that significant neuronal loss in this region was observed in only one of the KA-treated group, namely in rats that had experienced convulsive SE. However, it is possible that even small loss of CA3 neurons, perhaps, undetectable in small-size samples (such as those used in the present study), may have a substantial impact on hippocampal function. This possibility is consistent with accepted theoretical models of hippocampal circuits [56], [57], [58] and experimental studies [59], [60], [61] implicating the CA3 region of the hippocampus in key cognitive processes underlying episodic memory, such as pattern separation (reducing the overlap between similar neuronal representations) and pattern completion (converging neuronal representations into a single pattern, thus, facilitating their retrieval using only partial cues). Therefore, it is plausible that the memory deficits observed in KA-treated rats in this study can be accounted for, partly at least, by the disruption of cognitive processes in CA3 neuronal networks, which may be associated with the loss of neurons in this region.

Although in this study we have not estimated neuronal numbers separately for the dorsal and ventral parts of the hippocampal formation, visual inspection of the brain sections clearly showed that neuronal loss associated with behavioral SE was more marked in the ventral division of the dentate hilus than in its dorsal part (Fig. 6). This finding is in line with a previous study demonstrating that kainate-induced SE produces more severe loss at the temporal (ventral) pole of the dentate hilus when compared to the septal (dorsal) pole [28]. However, in the no-BSE group, the neurons of the ventral hilus were less severely affected. This observation is important for the interpretation of the finding that rats in the no-BSE group, unlike those that had experienced motor SE, showed normal behavior in the open-field, elevated plus-maze and fear-conditioning tests. Indeed, considerable evidence suggests that the ventral hippocampus has a preferential role in the regulation of emotional behaviors in rodents [49]. Therefore, the different extents of cell loss in the ventral hilus of rats from the BSE and no-BSE groups can be related to the differences in their performance on the anxiety-related tests. This explanation, however, should be viewed with caution given that brain regions other than hippocampus, e.g. the amygdala, play important roles in emotional behaviors and were not analyzed histologically in the present study. Another implication of this morphological observation is that the greater loss of neurons in the ventral dentate hilus of BSE rats may be associated with the increased susceptibility to the development of spontaneous motor seizures. Relevant to this hypothesis is the finding by Inostrosa et al [9], who showed that kainate-induced convulsive SE produces significant mossy fiber sprouting only in the ventral part of the dentate gyrus, suggesting an enhanced susceptibility of this hippocampal subdivision to epileptogenesis [28]. Furthermore, it has been previously reported that KA injection into the ventral hippocampus induces local electrographic seizures, which rapidly propagate to the dorsal hippocampus and cingulate cortex and are accompanied by severe motor manifestations including stage 4 and 5 seizures on the Racine scale [62]. In contrast, injection of KA into the dorsal hippocampus induces only local electrographic seizures, which do not propagate to the cingulate cortex and do not evoke behavioral seizures [62]. Therefore, it is possible that the absence of spontaneous motor seizures in no-BSE rats may be related to the fact that the ventral hippocampus was better preserved in this group when compared to the BSE group, in which all rats showed spontaneous motor seizures. This, of course, does not mean that the absence of convulsive SE after the treatment with KA precludes the development of epilepsy later in life. Indeed, because in the present study we did not perform EEG monitoring, spontaneous subconvulsive seizures might have occurred in the no-BSE group, but were not registered. In addition, the latency between the kainate treatment and the onset of the first spontaneous seizures may exceed the 5-month period of observation implemented in the current study [16].

In conclusion, this study was designed to compare the behavioral and neuroanatomical outcomes in rats that developed convulsive SE after kainate treatment to those that did not. It was found that those rats in which KA did not induce motor signs of SE were, however, impaired in hippocampal-dependent memory tasks later in life, which can be explained by long-term functional abnormalities in hippocampal circuits associated with the loss of hilar cells and, probably, CA3 pyramidal neurons. However, the extent of neuronal loss and the severity of cognitive deficits in this group were far less striking than those observed in the rats that had experienced motor SE. Thus, our findings add further weight to the evidence from former studies in animal models [9], [53] and humans [4], [6], [63] showing that behavioral deficits in TLE correlate with the extent of hippocampal damage.
